# *TARGET OF RAPAMYCIN* is essential for asexual vegetative reproduction in *Kalanchoë*

**DOI:** 10.1093/plphys/kiab589

**Published:** 2021-12-22

**Authors:** Kirsty McCready, Victoria Spencer, Francisco Jácome-Blásquez, Jamie Burnett, Itzel Margarita Viveros Sánchez, Zara Riches, Minsung Kim

**Affiliations:** School of Biological Sciences, Faculty of Biology, Medicine and Health, The University of Manchester, M13 9PT, UK; School of Biological Sciences, Faculty of Biology, Medicine and Health, The University of Manchester, M13 9PT, UK; School of Biological Sciences, Faculty of Biology, Medicine and Health, The University of Manchester, M13 9PT, UK; School of Biological Sciences, Faculty of Biology, Medicine and Health, The University of Manchester, M13 9PT, UK; School of Biological Sciences, Faculty of Biology, Medicine and Health, The University of Manchester, M13 9PT, UK; School of Biological Sciences, Faculty of Biology, Medicine and Health, The University of Manchester, M13 9PT, UK; School of Biological Sciences, Faculty of Biology, Medicine and Health, The University of Manchester, M13 9PT, UK

## Abstract

The unique mechanism by which leaf margin cells regain potency and then form a plantlet in *Kalanchoë* spp. remains elusive but involves organogenesis and embryogenesis in response to age, day length, nutrient availability, and drought stress. In light of this, we investigated whether TARGET OF RAPAMYCIN (TOR), a conserved protein kinase in eukaryotes that controls cell growth and metabolism in response to nutrient and energy availability, may regulate plantlet formation. *Kalanchoë daigremontiana TOR* (*KdTOR*) was expressed in the leaf margin at the site of plantlet initiation, in the early plantlet cotyledons, and in the root tip of the developed plantlet. Both chemical and genetic inhibition of TOR Kinase activity in *Kalanchoë daigremontiana* leaves disrupted plantlet formation. Furthermore, downregulation of *KdTOR* in transgenic plants led to wide-ranging transcriptional changes, including decreased *K. daigremontiana SHOOTMERISTEMLESS* and *K. daigremontiana LEAFYCOTYLEDON1* expression, whereas auxin treatments induced *KdTOR* expression in the plantlet roots. These results suggest that the *KdTOR* pathway controls plantlet development in cooperation with auxin, organogenesis, and embryogenesis pathways. The ancient and highly conserved TOR Kinase therefore controls diverse and unique developmental pathways, such as asexual reproduction within the land plant lineage.

## Teaser

The highly conserved eukaryotic nutrition-sensing regulator TARGET OF RAPAMYCIN (TOR) regulates a unique method of asexual reproduction in plants.

## Introduction

**Figure 1:**
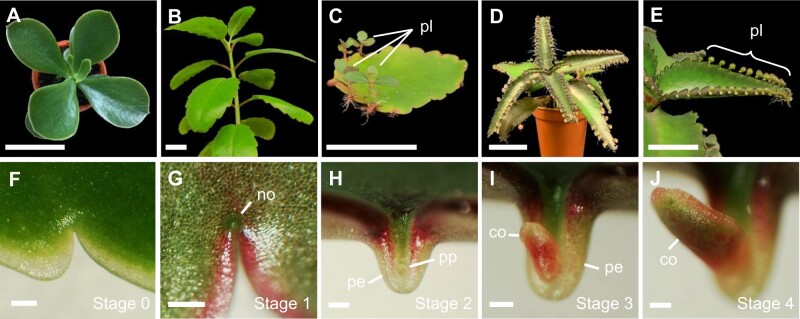
Plantlet formation in *Kalanchoë*. A–E, *Kalanchoë* species evolved different strategies in terms of plantlet formation. *Kalanchoë thyrsiflora* does not make plantlets (A), whereas *K. pinnata* makes plantlets when leaves are detached (B and C). *Kalanchoë daigremontiana* constitutively makes plantlets along the margins of the leaves in favorable conditions (D and E). F–J, Stages of WT *K. daigremontiana* plantlet formation. F, Stage 0: young leaf indentation with no evidence of pedestal or plantlet. G, Stage 1: no pedestal has formed and the margin is visibly raised at the node. H, Stage 2: a pedestal has formed. I, Stage 3: a thin, pin-shaped plantlet emerges from pedestal, which is visible with the naked eye. J, Stage 4: plantlet cotyledons begin to elongate and become rounder. Scale bars: 5 cm (A–E); 200 µm (F–J). co, cotyledon; no, node; pe, pedestal; pl, plantlets; pp, plantlet primordium.

Cell differentiation in multicellular organisms confers specialized functions to different tissue types. However, some plants have evolved the ability to reverse this process to regain totipotency. In several *Kalanchoë* spp. (Crassulaceae), mature leaf cells in the serrations along the leaf margins become totipotent, and form small clonal individuals (plantlets), which detach to form an individual plant (Batygina et al., 1996). Within the *Kalanchoë* genus, the triggers for this process vary, perhaps in response to the ecological contexts in which they evolved. Some species are unable to produce plantlets (*e.g*., *K. thyrsiflora*; Figure 1A); others produce plantlets upon stress induction (*e.g.*, *K. pinnata*; Figure 1, B and C); while some species produce plantlets constitutively in favorable conditions (*e.g.*, *K. daigremontiana*; Figure 1, D and E;  Garcês et al., 2007). Phylogenetic analyses suggest that lack of plantlets is the ancestral state, whereas inducible and constitutive plantlet formation are more derived (Garcês et al., 2007).

Most studies to unravel the molecular mechanisms behind plantlet formation have been performed with *K. daigremontiana* (Garcês et al., 2007, 2014; Liu et al., 2016; Zhu et al., 2017)*.* While a *K. daigremontiana* plant is capable of forming plantlets throughout its lifespan, the timing of this event is influenced by the age of the plant, the maturity of the leaves, and the environmental conditions such as day length and water availability (Liu et al., 2016). One potential mechanism for this is through the circadian clock gene, *SUPRESSOR OF OVEREXPRESSION OF CONSTANS 1* (*SOC1*), the expression of which corresponded with conditions that induced plantlets, such as long days and drought (Liu et al., 2016). Furthermore, the overexpression of *KdSOC1* reduced plantlet formation, and increased the expression of an auxin efflux carrier, *PINFORMED1* (*PIN1*), and auxin content in leaves (Zhu et al., 2017).

Once the appropriate conditions are met, plantlets are produced sequentially from the tip to the base of the leaf, within the serrations in the leaf margin (Johnson, 1934). These cells must convert from differentiated mature leaf cells, into stem cells, and organogenesis meristem maintenance genes have been shown to be involved in this process (Garcês et al., 2007). For example, *SHOOTMERISTEMLESS* (*STM*) is a *KNOTTED1-LIKE HOMEOBOX* (*KNOX*) gene in Arabidopsis (*Arabidopsis thaliana*) involved in the maintenance of stem cells in the shoot apical meristem (SAM; Endrizzi et al., 1996). *Kalanchoë* *daigremontiana STM* (*KdSTM*) was expressed in the SAM, the axillary buds, the initial cells that will form the plantlet and the upper part of the cotyledons of a heart-stage plantlet (Garcês et al., 2007). Furthermore, *KdSTM* RNAi lines prevented plantlet formation in *K. daigremontiana* (Garcês et al., 2007). Consistently, *STM* expression was absent in leaf margins of *Kalanchoë* species that do not produce plantlets (Garcês et al., 2007).

After plantlet initiation, constitutive plantlet development resembles zygotic embryogenesis, with clear globular and heart stages, and as such has been linked to genetic embryogenesis pathways (Garcês et al., 2007). *Arabidopsis* *thaliana LEAFY COTYLEDON1* (*LEC1*), *LEC2*, and *FUSCA3* (*FUS3*) are closely related embryogenesis genes that are expressed during embryo morphogenesis and maturation, and control reserve accumulation and desiccation tolerance of seeds (Braybrook and Harada 2008). Ectopic *LEC1* and *LEC2* expression in Arabidopsis can induce vegetative somatic embryo formation (Lotan et al., 1998; Stone et al., 2001), similar to plantlet formation in *Kalanchoë* (Garcês et al., 2007). In accordance with this, the *K. daigremontiana LEAFYCOTYLEDON1* (*KdLEC1*) homolog is expressed in the zygotic embryo and the heart-stage of developing plantlets (Garcês et al., 2007). However, *K. daigremontiana* has a truncated LEC1 protein, which cannot rescue the *lec1* mutant when expressed in *A. thaliana*, and is also unable to form viable seeds due to desiccation intolerance (Garcês et al., 2007). This suggests that truncated *LEC1* is required for constitutive plantlet formation, to bypass plantlet dormancy.

Although not fully elucidated, it is clear that *K. daigremontiana* plantlet formation requires complex signaling pathways integrating photoperiod, hormones, embryogenesis, and organogenesis. TARGET OF RAPAMYCIN (TOR) kinase is a highly conserved regulator of cell growth in response to nutrient and energy availability in eukaryotes (Dobrenel et al., 2016), and has been shown to integrate light and hormone availability to control embryogenesis and organogenesis in plants (Menand et al., 2002; Deprost et al., 2005; Moreau et al., 2012; Schepetilnikov et al., 2013; Pfeiffer et al., 2016; Wang et al., 2018). While TOR activity is controlled by the nutrient and energy availability in the cell in all eukaryotes (Dobrenel et al., 2016), the types of nutrients and energy sources in each lineage of eukaryotes vary. For example, TOR in plants is activated by glucose derived from photosynthesis (Xiong et al., 2013). Furthermore, plants independently evolved multicellularity, and have specialized cell types for nutrient acquisition (*e.g.*, leaves and roots) as well as their own hormone signaling mechanisms (Chaiwanon et al. 2016). Of these hormones, the “growth” hormone auxin activates TOR (Schepetilnikov et al., 2013; Li et al., 2017), while the “stress” hormone abscisic acid (ABA) inhibits TOR (Wang et al., 2018). Furthermore, the activation of TOR is tissue specific, as glucose and light are required for TOR activation in the shoot, but only glucose is required in the root (Xiong et al., 2013; Li et al., 2017).

Inducible *TOR* knockdown lines and chemical TOR inhibition have shown multiple developmental phenotypes, such as smaller leaves, reduced leaf number, delayed lifespan, increased branching, and flower sterility (Deprost et al., 2007; Mohammed et al., 2018), and *TOR* overexpression lines show the opposite phenotypes (Deprost et al., 2007; Ren et al., 2011). Auxin controls many of these processes, and TOR controls the translation of the auxin signaling genes, *AUXIN RESPONSE FACTORS* (*ARFs*; Schepetilnikov et al., 2013). In the presence of glucose but absence of light, exogenous application of the auxin, indole-3-acetic acid (IAA), activated TOR in the shoot apex (Li et al., 2017). Clearly, auxin is in a complex feedback loop with TOR to control development, by acting both upstream and downstream of TOR function.

TOR kinase also controls the expression of organogenesis genes, such as *WUSCHEL* (*WUS*; Pfeiffer et al. 2016). Pfeiffer et al. (2016) found that after 3 days’ incubation with the TOR inhibitor AZD-8055, TOR activity decreased, as did *WUS* expression. TOR has also been implicated in cellular dedifferentiation during callus formation in Arabidopsis tissue culture. By integrating sugar sensing and E2 PROMOTER-BINDING FACTOR a (E2Fa) phosphorylation, TOR drives transcriptional activation of S-phase genes for cell proliferation to make callus tissues (Lee and Seo, 2017). Furthermore, a study examining metabolic and hormonal profile shifts during *in vitro* organogenesis in tomato showed that *TOR* transcripts increased during callus formation (Kumari et al., 2017). Together, these experiments suggest a possible role for TOR in triggering pluripotency in differentiated somatic cells.

Due to TOR’s ability to sense hormone availability and environmental conditions to control embryogenesis and organogenesis, we investigated whether and how *Kalanchoë daigremontiana TOR* (*KdTOR*) is involved in plantlet formation. Here, we show that *KdTOR* is expressed during the early stages of plantlet development, and inhibition of its function disrupted plantlet formation, likely through *KdSTM* and *KdLEC1* downregulation. Furthermore, the plant hormone auxin is also involved in the *KdTOR* pathway in the plantlet roots. This work reveals the importance of *KdTOR* as a critical regulator of plantlet formation.

## Results

### *KdTOR* is expressed during plantlet initiation in the leaf margins

To investigate whether TOR was recruited for plantlet development in *Kalanchoë* leaves, reporter lines under the control of the *KdTOR* promoter and 5′-untranslated region (UTR) were generated. Three independent lines showing consistent β-glucuronidase (GUS) expression patterns were analyzed during plantlet development. Within the wild-type (WT) leaf indentations, four stages of plantlet development can be distinguished (Figure 1, F–J). Stage 1 is identified by a raised node at the indented region, with no protrusion or visible plantlet (Figure 1G). Stage 2 is defined by a pedestal, which is a protruding structure that will hold the developing plantlet (Figure 1H). Next, a visible, pin-shaped plantlet will form from the pedestal (stage 3; Figure 1I), before the anisocotylous plantlet cotyledons begin to round (stage 4; Figure 1J).

**Figure 2:**
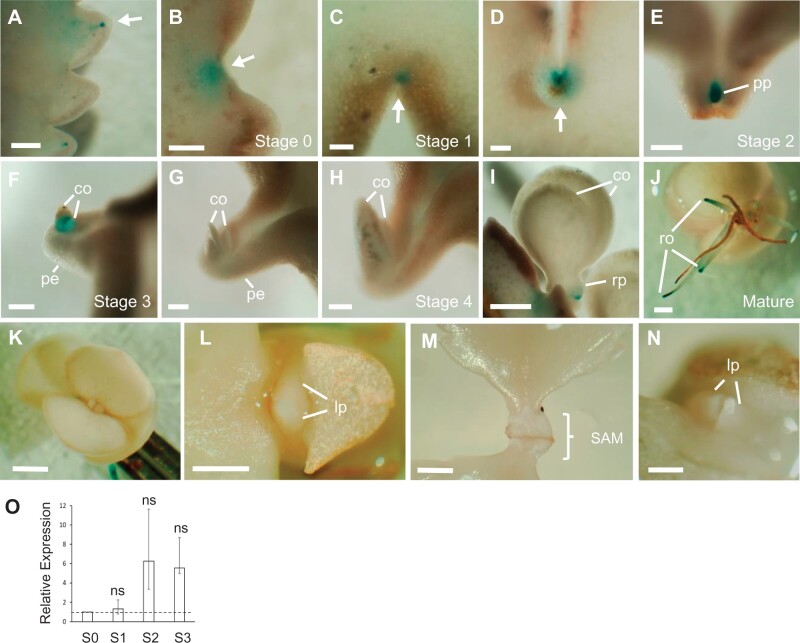
Expression of *KdTOR* through *K. daigremontiana* plantlet formation. A–G, *pKdTOR::GUS* lines showed *GUS* expression through stages 0–3 of plantlet formation. Signal was detected at the hydathode (A, arrow), the indentation of the leaf margin (B, arrow), and at the node within the indentation (C and D, arrows). At the pedestal, *GUS* was expressed in the initiating plantlet primordium (E) and in the early stages of cotyledon development (F), but not in the expanding cotyledons (G–I). While signal was detected in the root primordia (I) and the root tips of mature plantlets (J), GUS did not accumulate in the SAM of plantlets (K, L) or mature plants (M, N). O, RT-qPCR of *KdTOR* expression in stages 1–3, relative to stage 0. One-way ANOVA with Dunnet’s multiple comparison (*P* < 0.05; *n* = 3). Error bars show SEM. Scale bars: 1 mm (A, I, J, K, and M); 0.5 mm (L and N); 200 µm (B–H). co, cotyledon; pe, pedestal; pp, plantlet primordium; rp, root primordium; ro, root; lp, leaf primordium; SAM, shoot apical meristem.

*GUS* accumulated in the hydathodes of developing leaves (Figure 2A, arrow) and in the indentations before the onset of plantlet formation (stage 0; Figure 2B, arrow)*. GUS* expression was later detected at stage 1 indentations at the node (Figure 2, C and D, arrows). *GUS* expression was weak in the pedestal itself (stage 2; Figure 2E) but was strongly expressed in the plantlet primordium as it began to emerge (Figure 2E). The stage 3 pin-shaped plantlet showed a dramatic reduction in *GUS* expression (Figure 2F), compared to the initiating plantlet (Figure 2E), and it was often undetectable in late stage 3 plantlets (Figure 2G). The expression across the emerging plantlet was not homogeneous; the tip of the developing cotyledon had less *GUS* expression than the base (Figure 2F). No *GUS* expression was detected in the stage 4 plantlet cotyledons (Figure 2H). *GUS* expression was present in the root primordia of the developing plantlet (Figure 2I) and was later detected at the root tip of mature plantlets (Figure 2J). Conversely, no obvious expression was detected in the SAM or leaf primordia of the mature plantlet (Figure 2, K and L), nor in the SAM of the mature plant (Figure 2, M and N).

*pKdTOR::GUS* expression suggests that *KdTOR* is expressed in the early plantlet stages, so to confirm this for native *KdTOR* transcripts, *KdTOR* expression in stages 0–3 was quantified by reverse transcription quantitative polymerase chain reaction (RT-qPCR). *KdTOR* expression was detected at stages 0–3 of plantlet formation (Figure 2O). While RT-qPCR data were not statistically significant, the trend of the expression levels among plantlet developmental stages was comparable to those of *pKdTOR::GUS* data. This trend of *KdTOR* expression was repeatedly seen in several independent RT-qPCR experiments. Compared with stage 0, *KdTOR* expression did not markedly increase during stage 1 of plantlet formation, in agreement with *pKdTOR::GUS* lines, which have similar GUS intensities at these two stages. *KdTOR* expression in stages 2 and 3 increased compared with stages 0 and 1. RT-qPCR suggests that *KdTOR* may play a role in plantlet formation throughout the developmental stages, but is most crucial for the initiation of plantlet formation at the pedestal (stage 2), as also supported by strong *pKdTOR::GUS* expression.

### Torin2 and AZD-8055 reduced total plant growth and inhibited plantlet formation

**Figure 3:**
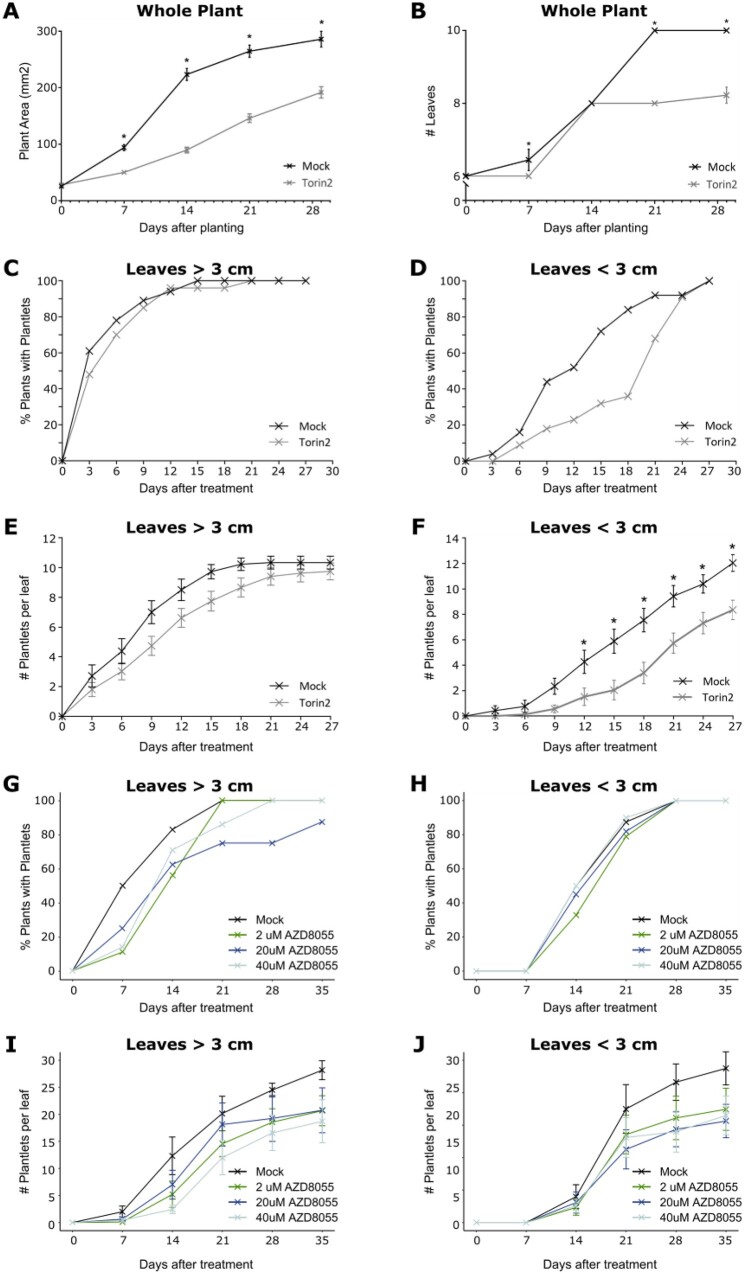
Torin2, AZD-8055, and mock treatment of *K. daigremontiana*. A, B, Area and leaf number of plantlets planted on media containing mock solution or 100-µM Torin2. C–F, 100-µM Torin2 brushed directly onto the leaf margins reduced plantlet formation (*n* = 25). G–J, 2-µM, 20-µM, and 40-µM AZD-8055 brushed directly onto the leaf margins reduced plantlet formation independent of leaf size (>3 cm, *n* = 32, <3 cm, *n* = 45). Error bars show SEM. Two-way ANOVA (repeated measures) with Sidak’s multiple comparisons tests (95% confidence limits: ns; *P ≤ 0.05; **P ≤ 0.01; ***P ≤ 0.001; ****P ≤ 0.0001) (A, B, E, and F) and least squares mean with Tukey’s *P*-value adjustment (95% confidence limits) (I and J).

To investigate whether *KdTOR* has conserved function in controlling growth and development in *K. daigremontiana*, plantlets were grown *in vitro* on media containing an ATP-competitive chemical inhibitor that has been shown to inhibit TOR, known as Torin2 (Liu et al., 2013; Figure 3, A and B). Plant area was significantly lower when grown on 100-µM Torin2 media compared to mock media (*P* = 0.0013, *t* = 3.831 [Day 7]; *P* < 0.0001, *t* = 11.68, 10.36, 8.222 [Days 14, 12, 28]; Figure 3A), implying that KdTOR controls plant growth in *Kalanchoë.* Torin2-treated plants also had significantly fewer leaves on Day 7 (*P* = 0.0419, *t* = 2.697), Day 21 (*P* < 0.0001, *t* = 12.14), and Day 29 (*P* < 0.0001, *t* = 10.79; Figure 3B).

To determine whether *KdTOR* is involved in plantlet formation, *K. daigremontiana* leaf margins of plants grown on soil were brushed with 100-µM Torin2 or mock solution. Due to the external method of application, we used a higher Torin2 concentration (100 µM) than previous studies (Montané and Menand, 2013), and the effects of this treatment were confined to plantlet formation without any visible side-effects in other parts of the leaf. First, for leaves >3cm, at all time points the percentage of plants with plantlets was similar when treated with mock or Torin2 solution (Figure 3C). However, leaves <3cm had a lower percentage of plants with plantlets between Days 3 and 24 when treated with Torin2 compared to mock (Figure 3D). Overall, Torin2-treated leaves of both sizes had significantly fewer plantlets (in leaves >3cm: *P* = 7.084e-06, *F* = 20.6754; in leaves <3cm: *P* = 1.159e-14, *F* = 63.6218; Figure 3, E and F), which was specifically lower from Day 12 (*P* = 0.0404, *t* = 2.884) through to the final day of measurement for leaves <3cm (Day 28; *P* = 0.0012, *t* = 3.873; Figure 3F). In younger leaves (<3cm), most indentations are stage 0 and plantlet formation is yet to initiate, whereas in older leaves (>3cm), the majority of indentations are stage 1 or 2 and therefore plantlet formation has already been triggered. By the end of the period (29 d after the Torin2 treatment), leaves were completely matured and no additional plantlets were formed, therefore fewer plantlets seen in Torin2-treated leaves were not due to a delay of leaf growth or plantlet initiation.

We further investigated the role of *KdTOR* in plantlet formation by applying a range of concentrations of ADZ-8055 (2 µM, 20 µM, and 40 µM) onto leaves, as ADZ-8055 targets and inhibits TOR more specifically than Torin2 (Chresta et al., 2010; Liu et al., 2013). Overall, AZD-8055 concentrations significantly reduced plantlet formation compared to the mock (in leaves >3cm: 2 µM, *P* = 0.0005584, *F* = 12.955; 20 µM, *P* = 0.0397, *F* = 4.388; 40 µM, *P* = 4.155e-05, *F* = 19.2924; in leaves <3cm: 2 µM, *P* = 0.03633, *F* = 4.5153; 20 µM, *P* = 0.004928, *F* = 8.2618; 40 µM, *P* = 0.01193, *F* = 6.5692; Figure 3, G–J). While higher concentrations broadly showed fewer plantlets (Figure 3, I and J), there was no statistically significant difference among the treatments at different concentrations. No noticeable side-effects other than plantlet formation were observed in AZD-8055-treated leaves (Supplemental Figure S1).

### *KdTOR* silencing lines showed defects in meristem patterning

**Figure 4:**
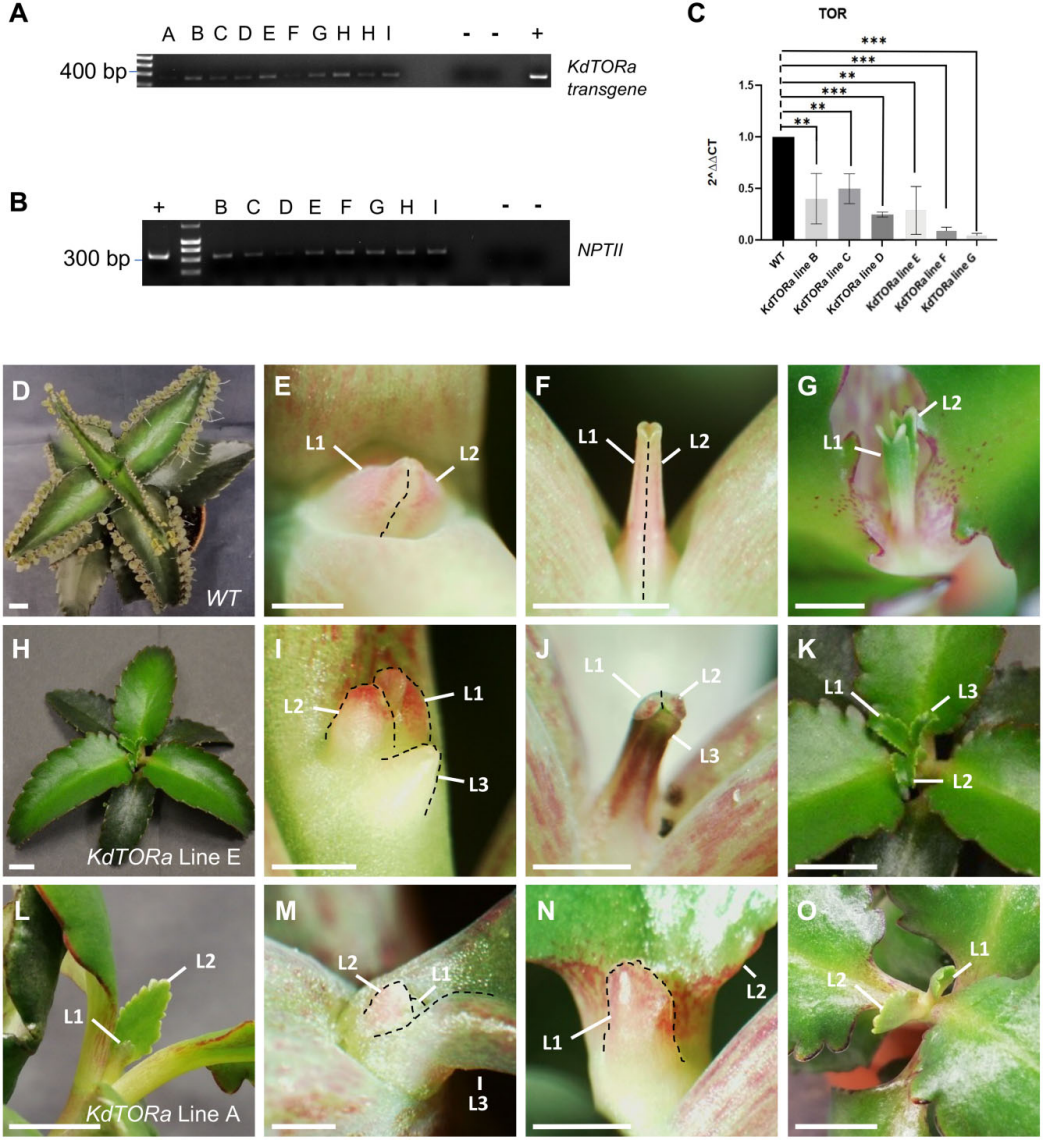
Genotypic analysis and phyllotaxy phenotypes of *35S::KdTORa* silencing lines*.* A–C, PCR confirms *35S::KdTORa* lines are transgenic and *KdTOR* is downregulated. Amplification of transgene (A) and *NPTII* (B), and RT-qPCR of *KdTOR* expression in independent *35S::KdTORa* lines (C). Negative control (−): WT; positive control (+): *KdTORa::pBI128* plasmid. *Kd18S* was used as a control for (C). One-way ANOVA with Dunnet’s multiple comparison, *n* = 3. ns; *P > 0.05; **P ≤ 0.01; ***P ≤ 0.001; ****P ≤ 0.0001. Error bars show SEM. D–O, Whole plant phenotypes in *35S::KdTORa* lines. D–G, WT *K. daigremontiana* leaves emerge in pairs in an opposite and decussate phyllotactic order (D). After the two leaf primordia emerge (L1 and L2, E), a hollow tube-like structure is formed (F), before the growth of the two equally sized leaves (G). H–K, *35S::KdTORa* leaves can emerge three at a time from the same node (H). Three similarly sized leaves can be detected from emergence to expansion (I–K). L–O, *35S::KdTORa* leaves can emerge in an alternate phyllotactic order (L), forming one leaf at a time (M). Consequently, the older leaves (L3, L2) are larger than the younger leaves (L1) (N and O). Scale bars: 1 cm (D, H, L, G, K, and O); 400 µm (E, I, M); 1 mm (F, J, N). L1; Leaf 1, L2; Leaf 2, L3; Leaf 3.

While informative, chemical inhibition is only transient and may have off target effects (Liu et al., 2013). Therefore *35S::KdTORa* silencing lines were generated to investigate how decreased endogenous *KdTOR* affects plantlet development. For the silencing lines, we amplified and used a 276-bp fragment of *KdTOR* exon 8 that corresponded to the HEAT repeat domain and showed high conservation across the plant kingdom (Supplemental Figure S2 and Supplemental Table S1). A total of nine independent *35S::KdTORa* silencing lines were confirmed by PCR (Figure 4, A and B) and downregulation of *KdTOR* expression was confirmed in eight lines (A–H) by RT-qPCR (Figure 4C) and/or semi-quantitative RT-PCR (Supplemental Figure S3). All eight lines showed substantial decreases in *KdTOR* expression compared to the WT (Figure 4C;  Supplemental Figure S3), suggesting that the suppression of *KdTOR* had been achieved. However, these lines did not show complete downregulation of *KdTOR*. This could be explained by the lethality of severe *KdTOR* knockdowns; only weaker knockdowns survived into adulthood, consistent with embryo lethality in *A. thaliana* (Menand et al., 2002; Deprost et al., 2007).

**Figure 5:**
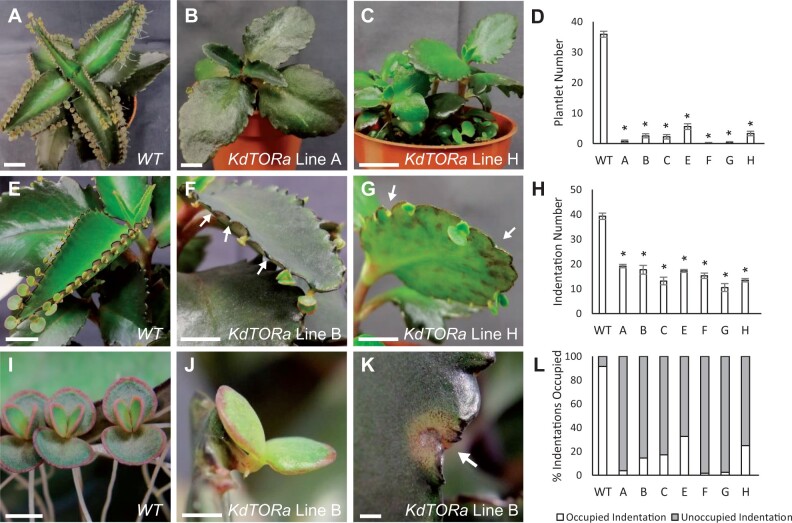
Phenotype analysis of plantlets in *K. daigremontiana 35S::KdTORa* silencing lines. A–D, *KdTOR* silencing significantly reduced plantlet formation. In WT *K. daigremontiana*, plantlets are produced along the leaf margins (A). As illustrated in lines A (B) and H (C), all transgenic lines had significantly fewer plantlets per leaf (*P* <0.0001) than the WT (D). E–H, *KdTOR* silencing significantly reduced indentation of the leaf margin. WT *K. daigremontiana* have regular leaf margin indentations where plantlets form (E). Margins of *KdTOR* silencing lines appeared smooth and irregular (F and G), and had significantly fewer indentations (*P* <0.0001) in the leaf margins than the WT (H). I–L, The indentations of *KdTOR* leaves were rarely occupied by a plantlet. In WT plants, nearly all indentations along the leaf margin are occupied by a plantlet (I), whereas transgenic plants have a lower percentage of indentations occupied (L) with a plantlet (J) than without (K). One-way ANOVA with Dunnett’s multiple comparison (*n* = 9). Error bars show SEM. Scale bars: 1 cm.

Phenotypes varied across lines, but a general reduction in whole plant and leaf size was observed (Figure 5, A–C), consistent with TOR repression in other species (Deprost et al., 2007; Xiong et al., 2016; De Vleesschauwer et al., 2018). The most prominent phenotypes observed in these transgenic lines were defects in meristem patterning. WT *K. daigremontiana* forms pairs of leaves in an opposite and decussate phyllotactic order; each pair of leaves is positioned at a 90° angle to the previous pair (Figure 4D). Two young equally sized leaf primordia emerge (Figure 4E) and form a hollow tube-like structure (Figure 4F), which grows into a pair of young and equally sized leaves (Figure 4G). In two independent lines (lines D and E), leaves instead emerged three at a time from the main meristem in at least 20% of individuals per line (Figure 4, H–K). After emergence (Figure 4I), an elongated tube structure consisting of three leaves develops (Figure 4J), before growth of the equally sized leaf blades (Figure 4K). Furthermore, leaves were produced in an alternate phyllotactic order in six independent lines (Figure 4L). At least 50% of individuals in lines B, C, and E, and at least 25% of individuals in lines D, F, and G produced leaves alternately, which were unequally sized throughout their development (Figure 4, M–O). In these lines, one leaf emerges at a time (Figure 4N), instead of a pair seen in the WT (Figure 4E). These changes to phyllotaxy suggest *KdTOR* may play a role in meristem patterning and leaf initiation in the mature plant, despite not being expressed directly in these tissues (Figure 2, M and N).

### *KdTOR* silencing lines have disrupted plantlet formation

All of the seven confirmed transgenic lines that were phenotyped had significantly fewer final plantlet numbers per mature leaf than WT plants (*P* < 0.0001; Figure 5, A–D), consistent with chemical leaf margin treatments (Figure 3, E, F, I, and J). Mean plantlet number was reduced by at least 85.5% (line E) to as much as 99.3% (line F) compared to the WT (Figure 5D). In some of the lines with the strongest silencing and most severe phenotypes (*e.g*., lines A and F), plantlet formation was nearly completely abolished (>99%; Figure 5, B–D). Indentation number was also significantly reduced in transgenic lines relative to the WT (*P* < 0.0001; Figure 5, E–H), by between 51% (line A) and 73% (line G) in all transgenic lines (Figure 5H). It could therefore be argued that plantlet formation is decreased due to the morphological alteration of the leaf margins in these lines. However, not all reduction of plantlets was due to the loss of indentations. Many normal-looking indentations had no plantlets in these transgenic lines (Figure 5, F, G, and K, arrows). Even taking into account the reduction in indentation number, the percentage of plantlets occupying each indentation is still markedly lower in these transgenic lines compared with the WT (Figure 5L). While on average 91% of WT indentations were occupied by a plantlet, in all transgenic lines only 1%–30% of indentations showed evidence of plantlet formation (Figure 5L). This suggests that a reduction in plantlets is not only due to decreased indentation formation, but also disruption to plantlet initiation.

**Figure 6:**
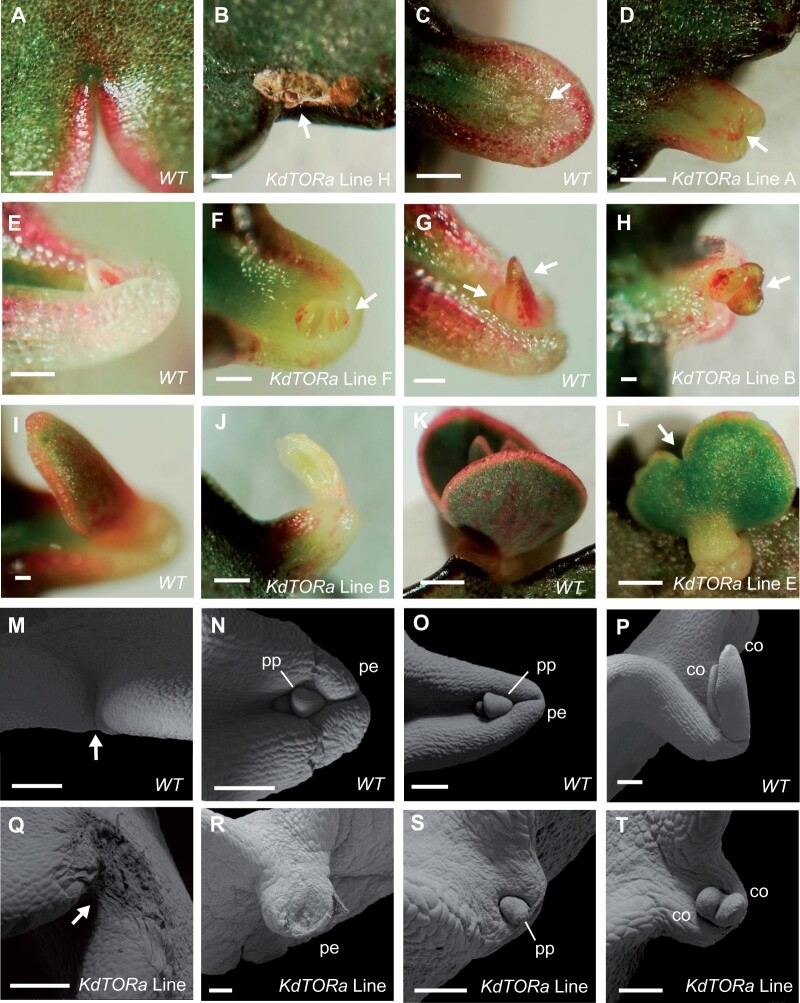
Defective *35S::KdTORa* plantlet development at the indentation. A–L, *KdTOR* silencing lines display defects in plantlet development at all stages of plantlet formation. The tissue rises at the node in the WT at stage 1 (A); however, in some *KdTOR* silencing lines, raised tissues at the node were aborted or became necrotic (B). Compared to the WT (C, E), the pedestal was misshapen and not visible (stage 2, D), or plantlet cotyledons emerging from the pedestal were misshapen and exposed on the shortened pedestal (stage 3, F) in transgenic lines. While the two WT cotyledons at stage 4 were beginning to round and stayed round (G, I, and K), the larger cotyledon of the transgenic plantlet often developed a bilobed shape (H). If the transgenic cotyledons did not become chlorotic and shrivelled (J), they retained their bilobed shape at maturity (L). M–T, SEM images of the WT (M–P) and *35S::KdTORa* indentations (Q–T), showing stage 1 (M and Q), stage 3 (N, O, R, and S), and stage 4 (P and T) plantlets. In transgenic plants, plantlet formation aborted prior to pedestal formation (Q), terminated at the pedestal, leaving necrotic tissue (R) or nonviable plantlet structures (S and T). Scale bars: 200 µm (A–J; M–T); 1 cm (K and L).

In some indentations, plantlets were not initiated at any stage of the plant’s lifespan; notches resembled stage 0 or terminated following the initial protrusion from the node and subsequent tissue necrosis (stage 1; Figures 5, K, 6, A and B, N, and Q, arrows). At most indentations, however, plantlet formation terminated following the formation of the pedestal (stage 2), which was often shorter, flattened and lacked the plantlet primordium (Figure 6, C and D, arrow, Figure 6, M and R). In this case, the termination of plantlet formation in *KdTOR* silencing lines was not simply delaying plantlet initiation, and it was rather a consequence of defective pedestals incapable of initiating plantlets. In the cases where plantlets were initiated, abnormal plantlet development was observed. Stage 3 plantlets were shorter and thicker, and more exposed on the pedestal, compared to the WT (Figure 6, E, F, S, and T). Stage 4 WT plantlet cotyledons are anisocotylous (Figure 6, G, O, and P, arrows). In some transgenic plants, the large macrocotyledon adopted a bilobed shape early in development (Figure 6H, arrow). At other stage 4 plantlets, leaves were contorted and bleached (Figure 6, I and J). Mature *35S::KdTORa* transgenic plantlet cotyledons were also discolored and bilobed (Figure 6, K and L). Unlike WT plantlets (Figure 5I), other mature plantlets had smaller, thicker, and misshapen leaves (Figure 5J), resembling the mother plants (Figure 5F). Overall, plantlet formation was terminated at each stage across the different lines and *KdTOR* is therefore likely to be critical for all initial stages of plantlet formation including pedestal development, plantlet primordium initiation, and morphological development. These suggested roles of *KdTOR* are also supported by *pKdTOR::GUS* expression analyses, in terms of spatio-temporal coincidence.

### Expression of key genes for plantlet formation were reduced in *KdTOR* silencing lines

**Figure 7:**
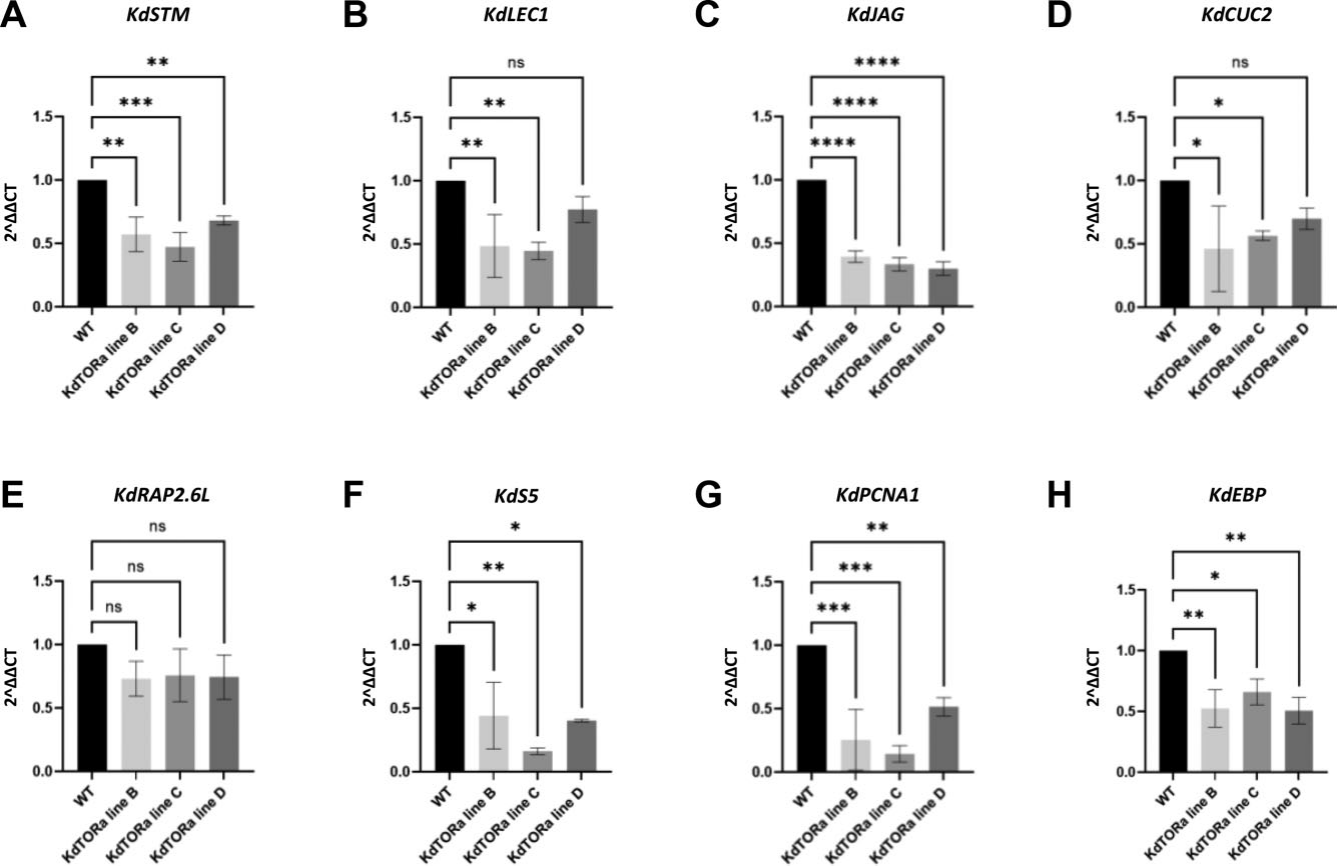
RT-qPCR analysis of *35S::KdTORa* silencing lines. A–D, RT-qPCR of *KdSTM, KdLEC1*, *KdJAG*, and *KdCUC2* expression in *35S::KdTORa* silencing lines, relative to the WT. E–H, RT-qPCR of known *TOR* downstream genes, *KdRAP2.6L*, *KdS5*, *KdPCNA1*, and *KdEBP*. *Kd18S* was used as the control. One-way ANOVA with Dunnet’s multiple comparison, *n* = 3. ns; *P > 0.05; **P ≤ 0.01; ***P ≤ 0.001; ****P ≤ 0.0001. Error bars show SEM.

In order to establish a genetic mechanism by which *KdTOR* may be regulating plantlet formation in stages 0–3, RT-qPCR was performed in *KdTOR* silencing lines to measure the expression of essential organogenesis (*KdSTM*) and embryogenesis (*KdLEC1*) genes. The expression of both *KdSTM* and *KdLEC1* was significantly lower than the WT (Figure 7, A and B), which suggests that failure to initiate plantlets in some leaf indentations may be due to the downregulation of *KdSTM* and *KdLEC1*. Furthermore, the expression of leaf crenulation genes *KdJAGGED* (*KdJAG*) and *KdCUP-SHAPED COTYLEDON 2* (*KdCUC2*) decreased in the transgenic lines (Figure 7, C and D). To confirm wide ranging transcription regulation by TOR, we analyzed genes, which have shown to be negatively (*RELATED TO AP2 6* [*AtRAP2.6L*]) and positively (*RIBOSOMAL PROTEIN S5* [*ATS5*], *PROLIFERATING CELLULAR NUCLEAR ANTIGEN 1* [*AtPCNA1*] and *ERBB-3 BINDING PROTEIN 1* [*AtEBP*]) regulated by TOR activity in published RNA Seq data sets (Xiong et al., 2013; Dong et al., 2015; Fu et al., 2021). While changes in *KdRAP2.6L* were not detectable, we confirmed expected the downregulation of *KdS5*, *KdPCNA1*, and *KdEBP* in response to decreased *KdTOR* expression (Figure 7, E–H).

### Auxin promotes *KdTOR* expression in the roots

**Figure 8:**
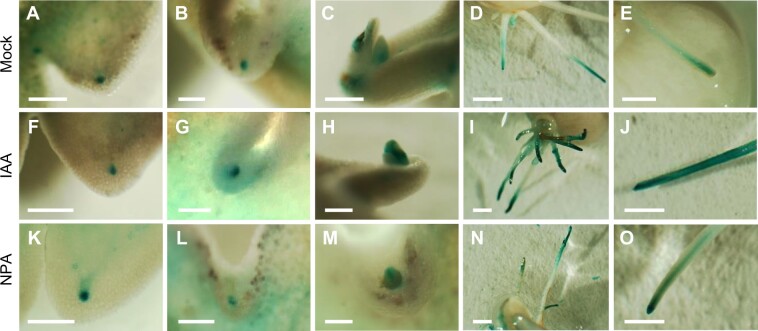
Auxin treatment of *pKdTOR::GUS* lines to test the upstream activation of *KdTOR*. *pKdTOR::GUS* leaves were incubated in mock solution (A–E), 25-µM IAA (F–J), or 25-µM NPA (K–O) for 24 h. There were no differences in expression in the hydathodes (stage 0; A, F, and K), in the plantlet primordia (stage 2; B, G, and L), or in the developing cotyledons (stage 3; C, H, and M). However, the roots of plantlets treated with IAA had stronger *GUS* expression in the epidermis and cortex of the elongation zone (I and J) when compared to mock (D and E) or NPA-treated (N and O) roots. Scale bars: 500 µm (A, F, and K); 100 µm (B, G, and L); 200 µm (C–E, H–J, and M–O).

To investigate whether auxin controls *KdTOR* expression, as it does TOR activity in Arabidopsis (Schepetilnikov et al., 2013; Li et al., 2017; Chen et al., 2018), leaves from *pKdTOR::GUS* lines generated here were incubated in auxin (IAA) and auxin transport inhibitor (naphthylphthalamic acid, NPA) solutions. In the hydathode (stage 0), *GUS* expression was not noticeably different between mock, 25-µM IAA, or 25-µM NPA treatments (Figure 8, A, F, and K). Similarly, the exposure to IAA or NPA did not affect the expression in the plantlet primordia (stage 2; Figure 8, B, G, and L) or the developing plantlet cotyledons (stage 3; Figure 8, C, H, and M). However, plantlet roots treated with IAA had noticeably higher *GUS* expression than mock or NPA-treated plants (Figure 8, D, E, I, J, N, and O). While *GUS* expression seemed to be localized to the division zone and internal cells of the elongation zone in the mock and NPA-treated plants, IAA-treated roots also expressed *GUS* in the epidermis and cortex of the elongation zone. This suggests auxin promotes *KdTOR* expression in the root, but not other tissues investigated.

## Discussion

*Kalanchoë* *daigremontiana* plantlet formation is a rare phenomenon in which the ability to regain potency has been exploited in plantlet development to reproduce asexually by creating clones (Garcês and Sinha, 2009a). In some *Kalanchoë* species, this method of reproduction is dependent on the environmental conditions in which the plant grows; plantlet initiation in *K. daigremontiana* is promoted by long days and drought (Liu et al., 2016). After initiation, plantlet development involves a combination of organogenesis pathways including *KdSTM* (Garcês et al., 2007), and embryogenesis pathways including *KdLEC1* (Garcês et al., 2007, 2014). Recently, TOR Kinase has emerged as a central player in controlling many aspects of plant development in response to energy and nutrient availability (McCready et al., 2020), as determined in part by environmental conditions. TOR is known to control plant embryogenesis (Menand et al., 2002), promote auxin signaling through *ARF* translation (Schepetilnikov et al., 2013), control meristem function through *WUS* and *YET ANOTHER KINASE 1* (*YAK1*; Pfeiffer et al., 2016; Barrada et al., 2019), and is itself promoted by light, glucose and auxin signaling in the shoot apices (Xiong et al., 2013; Li et al., 2017). Based on their similar reliance on environmental cues and embryogenesis and organogenesis signaling, we investigated the link between TOR kinase and plantlet formation.

Conservation of TOR Kinase amongst eukaryotes highlights its importance in growth and development across the plant and animal kingdoms. TOR phylogenies show protein sequence conservation and therefore possible functional conservation across the land plants and within *Kalanchoë* species (Sapre et al., 2018). Consistent with other eukaryotes, TOR controls cell growth and anabolism in plants (Dobrenel et al., 2016), and all TOR inhibition studies show a reduction in total plant size (Montané and Menand, 2013; Dong et al., 2015; Li et al., 2015, 2017; Xiong et al., 2016). Consistently, growing *K. daigremontiana* plants on the ATP-competitive chemical inhibitor, Torin2, reduced total leaf area. Arabidopsis *tor* knockdown lines also have fewer leaves (Deprost et al., 2007), as *AtTOR* controls leaf initiation by inhibiting cell cycle in response to glucose and light activation (Mohammed et al., 2018). We showed that the total leaf number was reduced after growing *K. daigremontiana* plants on Torin2, and so KdTOR may similarly be functioning to control *Kalanchoë* leaf initiation.

After establishing that *KdTOR* may have a conserved role in controlling plant size and leaf initiation, the relevance of *KdTOR* in plantlet formation was investigated. Chemical inhibition of KdTOR (with both Torin2 and AZD-8055) and *K. daigremontiana KdTOR* knockdown lines displayed a significant reduction in the number of plantlets produced along the leaf margins. This was due to failure to initiate at indentations, as well as plantlet termination at early stages (0–3), rather than delayed initiation, suggesting that *KdTOR* is essential for the initiation and growth of the plantlet from the pedestal. Correspondingly, *pKdTOR::GUS* transgenic lines and RT-qPCR revealed that *KdTOR* was indeed strongly expressed through these early stages, in agreement with *A. thaliana AtTOR* expression in the developing embryo (Menand et al., 2002). Previous RT-PCR studies showed that *AtTOR* mRNA was detectable in many tissue types (Robaglia et al., 2004), while *pTOR::TOR-GUS* line expression was specific to dividing cells (Menand et al., 2002). This suggests post-transcriptional regulation of *TOR* expression, and therefore our plantlet-specific expression may be due to post-transcriptional regulation of our GUS transcripts, perhaps due to the inclusion of the 5′-UTR in our lines.

Furthermore, *KdSTM* is an important regulator of stem cell identity and is necessary for inducing plantlet formation in the leaf margins (Garcês et al., 2007). RT-qPCR experiments here suggest that decreased *KdSTM* expression could be responsible for plantlet disruption in *KdTOR* silencing lines, perhaps through inability to trigger pluripotency in the leaf margins at stages 0 and 1. Similarly, *KdLEC1*, which is important for the embryogenesis-like progression of the initiating plantlet (Garcês et al., 2014), also had reduced the expression in *KdTOR* silencing lines. These data imply that in favorable conditions, TOR may be promoting *KdSTM* and *KdLEC1* expression for plantlet formation. We therefore provide evidence that *KdTOR* may be acting as the central regulator connecting nutrition sensing with the activation of downstream organogenesis and embryogenesis pathways. *KdJAG* and *KdCUC2* were also downregulated in *KdTOR* antisense lines. *JAG* and *CUC2* are key regulators controlling leaf crenulation (Dinneny et al., 2004; Nikovics et al., 2006), and smooth leaf margins in *KdTOR* lines might suggest *KdTOR* is involved in the leaf crenulation pathway through *KdJAG* and *KdCUC2*. Notably, our finding that *KdSTM*, *KdLEC1*, and *KdJAG* expression decreased in *KdTOR* transgenic lines contrasts to the situation in Arabidopsis where *TOR* does not regulate *STM*, *LEC1*, and *JAG* (Xiong et al., 2013; Dong et al., 2015; Fu et al., 2021). This suggests that *KdTOR* may have acquired unique downstream targets in the leaf to accommodate plantlet formation. We also investigated the expression levels of *TOR* downstream genes *KdS5*, *KdPCNA1*, *KdEBP*, and *KdRAP2.6L* in *KdTOR* plants. In Arabidopsis, inhibition of *TOR* decreased the expression levels of *S5*, *PCNA1* and *EBP* but increased *RAP2.6L* (Xiong et al., 2013; Dong et al., 2015; Fu et al., 2021). Similarly, downregulation of *KdTOR* decreased *KdS5*, *KdPCNA1*, *KdEBP* but did not affect the *KdRAP2.6L* level*.* This suggests that *KdTOR* is likely to retain the regulatory role in metabolic pathways in the *Kalancho*ë leaf, similar to that of Arabidopsis.

Alongside a reduction in plantlet number, changes to plantlet morphology were also observed at all developmental stages. The plantlet cotyledons of *KdTORa* silencing lines were misshapen and white in color, suggesting a loss of chlorophyll production. This is consistent with previous studies in Arabidopsis, in which TOR activity promoted chlorophyll biosynthesis during cotyledon greening through ABA-INSENSITIVE 4 (ABI4; Li et al., 2015). However, *pKdTOR::GUS* expression was not detected beyond stage 3 cotyledons. Notably, our data showed that some defective phenotypes in *KdTOR* transgenic plants manifested in a region where *pKdTOR::GUS* expression was absent, suggesting that some *KdTOR* actions may be cell nonautonomous. For example, altered phyllotaxy was observed in the absence of *GUS* expression in the SAM. This mismatching expression can also be seen in some Arabidopsis phenotypes; the expression of an *AtTOR::GUS* fusion protein was strongest in the *A. thaliana* embryo, the SAM and flower buds, but was absent from differentiating tissue such as expanding leaves (Menand et al., 2002), in which many *tor* phenotypes such as reduced leaf size and chlorophyll reduction are observed (Deprost et al., 2007). It will be informative to determine if the KdTOR protein or transcript is mobile, or is controlling indirect downstream signaling in distant tissues, despite the absence of *in situ* expression in our *pKdTOR::GUS* lines.

*pKdTOR::GUS* expression did however occur strongly in the root primordia of the developing plantlet, and was consistently detected at the tip of the growing root. *AtTOR* expression in root meristems has previously been reported in *A. thaliana* (Menand et al., 2002), and the recent discovery that *YAK1* is negatively regulated by TOR to promote root meristem maintenance (Barrada et al., 2019) provides scope for investigation of TOR as a conserved regulator of root meristem genes. In addition, *KdTOR* was expressed at the hydathode of the leaf. Similar *GUS* expression patterns in hydathodes and root tips are present in the auxin signaling reporter *DR5::GFP* Arabidopsis lines (Bilsborough et al., 2011), indicating that auxin and TOR may be interacting and this auxin localization is conserved in *Kalanchoë*. Changes to phyllotaxy in the main meristem of *KdTORa* lines also support the suggestion that *KdTOR* may be involved in downstream auxin signaling. Furthermore, we show that auxin promoted *KdTOR* expression in the plantlet root but not in the hydathode or the plantlet primordium. This tissue-specific *TOR* expression is reminiscent of TOR protein activity in Arabidopsis; while auxin-Rho of plants signaling activated TOR to trigger S phase in the SAM, auxin was not implicated in the activation of AtTOR-E2Fa signaling to activate the root apical meristem (Xiong et al., 2013; Li et al., 2017). It is important to note that the results presented here show only the changes in *KdTOR* gene expression, not protein activity, and it is possible that auxin activation of the KdTOR protein may be occurring in the hydathode or the plantlet, as it does in Arabidopsis (Chen et al., 2018). Auxin is also known to activate *AtTOR* to promote selective translation of *ARF* genes (Schepetilnikov et al., 2013; Li et al., 2017), so determining if this signaling module is conserved in *Kalanchoë* may elucidate the auxin–TOR–plantlet signaling pathway.

In conclusion, we have demonstrated that TOR’s conserved role as central mediator of environmental signals and developmental responses extends to the unique process of *K. daigremontiana* plantlet formation. How directly TOR controls developmental genes remains to be determined, both in *K. daigremontiana* and *A. thaliana*. Asexual reproduction in *Kalanchoë* species represents a unique innovation, requiring the reversion of differentiated cells to a totipotent state and recruitment of organogenesis and embryogenesis regulators. The confirmation that TOR signaling plays a key role in this process demonstrates how a conserved eukaryotic signaling pathway has been adopted for a novel Kalanchoë-specific process. As an ancient and robust signaling mechanism, TOR may have been recruited to integrate myriad environmental and nutritional information to ensure timely plantlet formation under favorable conditions, for optimal asexual reproduction. Due to their sessile nature, plants have evolved incredible resilience to alter their developmental and metabolic pathways in response to nutrient and energy availability. The *TOR* pathway plays a pivotal role during these developmental events and has contributed to the remarkable diversity within the plant kingdom.

## Materials and methods

### Plant materials and growth conditions

WT and transgenic *K.* *daigremontiana* plantlets were potted into a mix of Levington’s F2 compost (Scott’s Miracle Gro, UK), Perlite (Sinclair Horticulture Ltd, UK), and Vermiculite (Sinclair Horticulture Ltd, UK) in a 6:1:1 ratio and grown in an MLR-350 Versatile Environmental Test Chamber (Sanyo, Japan) in long day conditions (16-h light, 8-h dark, 680 LUX) at 23°C.

### Treatment conditions

Mature plantlets were taken from leaves and sterilized for 3 min in 20% (v/v) sodium hypochlorite and 0.001% (v/v) Triton X-100 (Fisher Scientific, UK). One plantlet per well was grown in six-well plates on half-strength Murashige and Skoog media (Duchefa Biochemie, The Netherlands) containing either 100-µM Torin2 (Sigma-Aldrich, UK) and 0.5% (v/v) DMSO (Sigma Aldrich-UK) or just 0.5% (v/v) DMSO (mock). Plant area was recorded every week for 4 weeks.

To treat *K. daigremontiana* leaves, plantlets were potted and grown for 6 weeks. Torin2 solutions (100-µM Torin2, 0.5% [v/v] DMSO, and 0.5% [v/v] Tween-20 [AppliChem, USA]) or AZD-8055 solutions (2 µM, 20 µM, or 40 µM AZD-8055 [Selleckchem, USA], 0.5% [v/v] DMSO and 0.5% [v/v] Tween-20 [AppliChem, USA]) and mock solutions (0.5% [v/v] DMSO and 0.5% [v/v] Tween-20) were applied by brushing the margins of plastochron 2 (P_2_) leaves. Leaf size was recorded before application, and plantlet number was recorded every 3 d for 27 d (Torin2 treatment) or every 7 for 35 d (AZD-8055 treatment). Plantlets were counted along one margin of one leaf per plant.

To treat *pKdTOR::GUS* lines, leaves of P_2_ and mature plantlets from three independent lines were incubated in IAA solutions (25-µM IAA [Sigma-Aldrich, UK], 0.1% [v/v] DMSO and 0.5% [v/v] Tween-20), NPA solutions (25-µM NPA [Fluka Analytical, Switzerland], 0.1% [v/v] DMSO, and 0.5% [v/v] Tween-20), and mock solutions (0.1% [v/v] DMSO and 0.5% [v/v] Tween-20) for 24 h. Treated leaves were then transferred to GUS staining solution.

### GUS staining

*pKdTOR::GUS* leaf margins and mature plantlets were incubated in GUS staining solution (100-mM sodium phosphate buffer, pH 7.2 [BDH Chemicals, UK], 10-mM EDTA, pH 8 [Promega, USA], 0.1% [v/v] Triton X-100 [Fisher Scientific, UK], 1-mM potassium ferricyanide (III) [Sigma-Aldrich, UK], 1-mM potassium ferrocyanide [Sigma-Aldrich, UK], 2-mM X-GlcA [Melford, UK]). Tissues were incubated in the dark for 24 h, and then cleared in 100% (v/v) ethanol.

### Gene cloning and vector assembly

Degenerate primers for *KdTOR* were designed against aligned *K. laxiflora* and *K. fedtschenkoi* sequences (obtained from Phytozome v12.1, JGI, University of California). For *KdTOR* antisense constructs, a 276-bp fragment of *KdTOR* exon 8 was cloned using gene specific primers (Supplemental Table S2). The primers for the *KdTOR* promoter fragment were designed to amplify 1,466-bp upstream of the start codon including the entire 5′-UTR region (Supplemental Table S2). The *KdTOR* exon 8 and promoter sequences were cloned using Q5 High Fidelity DNA Polymerase (New England Biolabs, USA) then ligated into pGEM-T Easy (Promega, USA) after gel extraction (Nucleospin gel and PCR Clean-Up Kit; Macherey-Nagel, Germany). Using Golden Gate assembly, the *KdTOR* exon 8 fragment was ligated in an antisense orientation with the cauliflower mosaic virus 35S Promoter and Terminator into a modified *pBI121* vector (*35S::KdTORa*). The promoter fragment of *KdTOR* was assembled with the coding region of *GU*S and the *Nopaline Synthase* Terminator into the modified *pBI121* vector (*pKdTOR::GUS*). Ligated constructs were then transformed in *Escherichia coli* strain *DH5α* for selection. Once confirmed, correct constructs were transformed into *Agrobacterium tumefaciens* strain *LBA4404* by electroporation and checked with culture PCR (Supplemental Table S2).

#### Kalanchoë daigremontiana *transformation*

WT *K. daigremontiana* plants were transformed with *35S::KdTORa* or *pKdTOR::GUS* as previously described (Garcês and Sinha, 2009b).

### Genotyping and phenotyping transgenic lines

DNA was extracted according to the “Quick DNA prep for PCR” protocol (Weigel and Glazebrook, 2002). PCR was performed with Q5 High-Fidelity DNA polymerase and BioTaq polymerase (Bioline, UK) and *KdTORa* and *35STerm* reverse primers (Supplemental Table S2). Additional PCR checks were carried out with NPTII forward and reverse primers (Supplemental Table S2). Cycling conditions were set according to the Q5 protocol, with annealing temperature of 58°C and extension for 30 s.

Plantlet number and indentation number of each transgenic line were recorded at leaf maturity (∼5 weeks). No further plantlets were formed after this time period.

### RNA extraction and cDNA synthesis

The indented notches of WT *K. daigremontiana* leaves at each plantlet formation stage (stages 0–3) and P1 leaves of individual *35S::KdTORa* lines (stage 0) were excised and frozen in liquid nitrogen. Several notches of the same stage were harvested from different leaves and grouped together into one sample to have enough tissue for RNA extraction. For *KdSTM* and K*dLEC1* RT-qPCR, the margins of newly emerging leaves (P1) were harvested. Total RNA was extracted with the RNeasy Plant Mini Kit (Qiagen, USA), using 10-mg polyvinylpyrrolidone (PVP, MW = 40,000) dissolved in 600-µL RLC Buffer per 100 mg ground tissue. RQ1 DNase (Promega, USA) and Tetro cDNA Synthesis (Bioline, UK) kits were used, according to the manufacturers’ protocols. cDNA synthesis reactions proceeded for 1 h at 45°C using a mixture of Random Hexamer and Oligo d(T) primers (Bioline, UK).

### RT-qPCR and RT-PCR

For RT-qPCR, a StepOnePlus Real-Time PCR machine with StepOne Software v2.3 was used, with a SensiFAST SYBR Hi ROX Kit (Bioline, UK). *GLYCERALDEHYDE-3-PHOSPHATE DEHYDROGENASE* (*KdGAPDH*) and 18S ribosomal RNA (*Kd18S*) were used as control genes (Supplemental Table S2; Garcês et al., 2014) with an annealing temperature of 60°C. The expression of these genes did not change in RNA Seq data sets Xiong et al., 2013, Dong et al., 2013 and Fu et al., 2021. Three biological replicates and three technical replicates were used; three independent lines were chosen, total RNA was extracted from three plants per line (nine plants in total), and the cDNA of each plant was tested three times for RT-qPCR. *qKdTOR* primers (Supplemental Table S3) were designed in exons 35 and 36, respectively. The comparative CT method was used for analysis.

For RT-PCR, 1X NH_4_ Reaction Buffer, 1.5-mM MgCl_2_, 1-mM dNTPs, 1-mM forward and reverse primers (Supplemental Table S3), 2.5 ng·µL^−1^ cDNA, 10 µL·mL^−1^ BioTaq polymerase and 10 µL·mL^−1^ Q5 High-Fidelity Polymerase were mixed in a final volume of 20 µL. A thermal cycling reaction was run according to settings recommended by the BioTaq protocol, with annealing at 58°C and extension for 30 s, for 39 cycles. *GAPDH* was used as a loading control and identical settings were used for 35 cycles.

### Image acquisition and data analysis

Photographs of *K. daigremontiana* were taken using a Huawei P smart (FIG-LX1) with an Apexel 10x Macro camera attachment. A GXCAM Eclipse (0654) Wi-Fi camera attached to a S8AP0 Stereo Microscope (Leica, USA) was used to visualize notches. Fiji Image J (http://imagej.net/Fiji/Downloads) was used to calculate plant areas and add scale bars. All graphs and statistical analyses were produced using GraphPad Prism Version 8.41. Two-way analysis of variance (ANOVA) (Repeated Measures) with Sidak’s multiple comparisons tests (95% confidence limits) were performed on the Torin2 treatment data. One-way ANOVA with Dunnett’s multiple comparisons tests were performed on the *35S::KdTORa* plantlet data.

### Scanning electron microscopy analysis

*Kalanchoë* *daigremontiana* leaves with plantlets at different developmental stages were fixed for scanning electron microscopy and viewed as described previously (Garcês et al., 2016; Zoulias et al., 2019).

### Phylogenetic tree construction

After sequencing the 276-bp *KdTOR* exon 8 fragment, the predicted *K. daigremontiana* peptide sequence (92 amino acids) was aligned with full length TOR peptide sequences from 38 other eukaryotic species. To obtain *TOR* orthologs for this alignment, a tBLASTn search was performed using the *A.* *thaliana* TOR peptide sequence (NP_175425.2) as a query. Where possible, the Reference RNA Sequences (refseq_rna) database on NCBI BLAST was used with default parameters (https://blast.ncbi.nlm.nih.gov/Blast.cgi?PROGRAM=tblastnPAGE_TYPE=BlastSearchLINK_LOC=blasthome). If species were absent from this list, a tBLASTn search was performed in Phytozome against their genomic databases with default parameters (https://phytozome.jgi.doe.gov/pz/portal.html#!search?show=BLAST). Species names, sequence accessions, databases used, and dates accessed can be found in Supplemental Table S1. Peptide sequences were aligned using MUSCLE with default parameters in MEGA X 10.1 software for macOS (Kumar et al., 2018; Stecher et al., 2020). The aligned sequences were trimmed to the 92 amino acid region homologous to the *Kd*TOR fragment before performing a maximum likelihood test using all sites to predict the best fit model for phylogenetic analysis. Based on these results, a maximum likelihood tree with a Jones–Taylor–Thornton model and Gamma (G) distributed substitution rate was constructed in MEGA X. The tree was rooted on the *Mus musculus* TOR peptide sequence. Any sequences that fell outside of the plant TOR monophyly were removed as they are unlikely to be TOR homologs. Five-hundred bootstrap replicates were performed.

### Accession numbers

The 276-bp *KdTOR* exon 8 fragment has the GenBank accession number MT955591. GenBank accession numbers of Arabidopsis orthologs of the genes used for RT-qPCR in Supplemental Table S3.

## Supplemental data

The following materials are available in the online version of this article.

**Supplemental Figure S1**. Plantlet formation 35 d after AZD-8055 treatments.

**Supplemental Figure S2**. Alignment and phylogeny of KdTOR with divergent plant species.

**Supplemental Figure S3**. Semi-quantitative RT-PCR in *TOR* antisense lines.

**Supplemental Table S1**. Sampling strategy for TOR phylogenetic tree construction.

**Supplemental Table S2**. List of primers used for gene cloning.

**Supplemental Table S3**. List of primers used for genotyping and RT-qPCR.

## Supplementary Material

kiab589_Supplementary_Data

## Notes

M.K. and V.S. designed the project. V.S., K.M., F.J-B., J.B., I.M.V.S., and Z.R. conducted the experiments. V.S. and K.M. generated transgenic lines, which were verified and analyzed by K.M. V.S. produced the phylogenetic tree. Z.R. performed Torin2 whole plantlet treatment experiments, while K.M. performed leaf margin Torin2 treatments, and J.B. and I.M.V.S. performed AZD-8055 treatment experiments. F.J-B., J.B., and I.M.V.S. performed RT-qPCR. F.J-B. performed *pTOR::GUS* treatments and staining, and some phenotypic analyses of the transgenic lines. V.S. and K.M. drafted and M.K. revised the manuscript.

The author responsible for distribution of materials integral to the findings presented in this article in accordance with the policy described in the Instructions for Authors (https://academic.oup.com/plphys/pages/general-instructions) is: Minsung Kim (minsung.kim@manchester.ac.uk).
