# Supplementary material for: TARGET OF RAPAMYCIN is essential for asexual vegetative reproduction in Kalanchoë
Source: Plant Physiol. 2021 Dec 22;189(1):248–63. doi: 10.1093/plphys/kiab589 (PMC9070829; doi:10.1093/plphys/kiab589)
Supplement: kiab589_Supplementary_Data [file kiab589_supplementary_data.pdf]

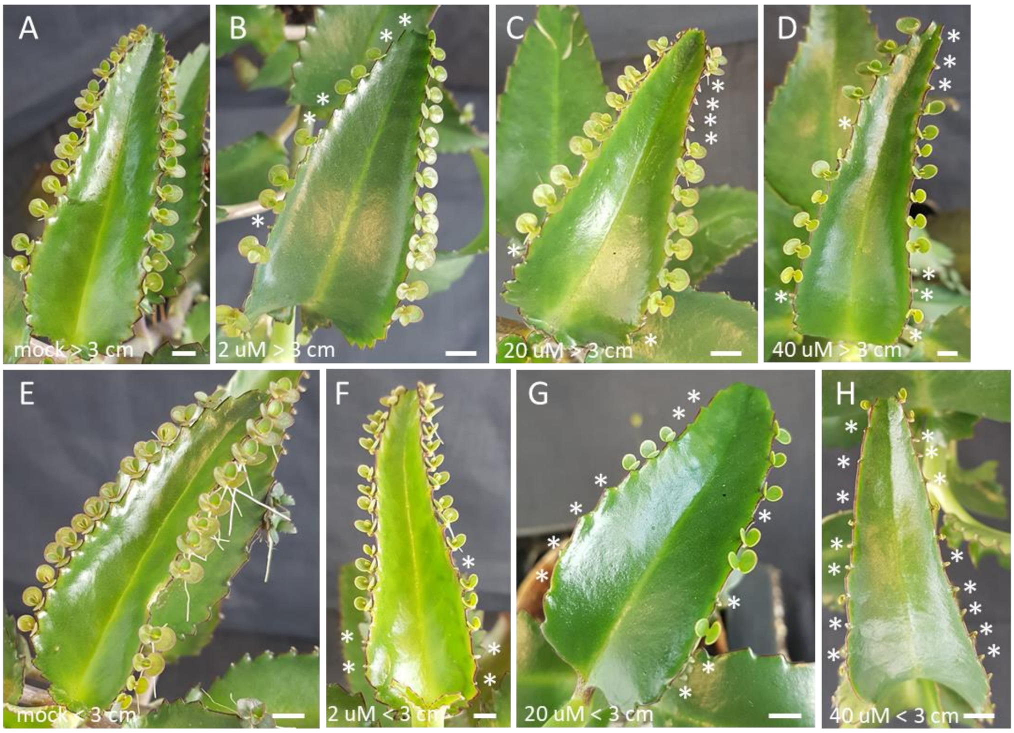

Supplemental Figure S1. Plantlet formation 35 days after AZD-8055 treatments. AZD-8055 in a range of concentrations, 0 µM (A, E), 2 µM (B, F), 20 µM (C, G) or 40 µM (D, H) was applied to developing leaves, bigger (A-D) or smaller (E-H) than 3 cm. Plantlet numbers were dramatically reduced in treated leaves. An indentation without any plantlet is marked by an asterisk. Scale bar: 0.5 cm

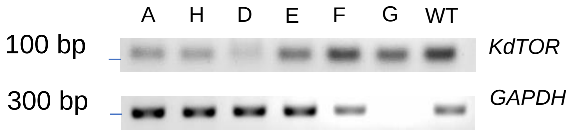

Supplemental Figure S3. Semi-quantitative RT-PCR in *TOR* antisense lines. *KdTOR* expression was reduced in independent *35S::KdTORa* lines. *KdGAPDH* was used as a control.

A

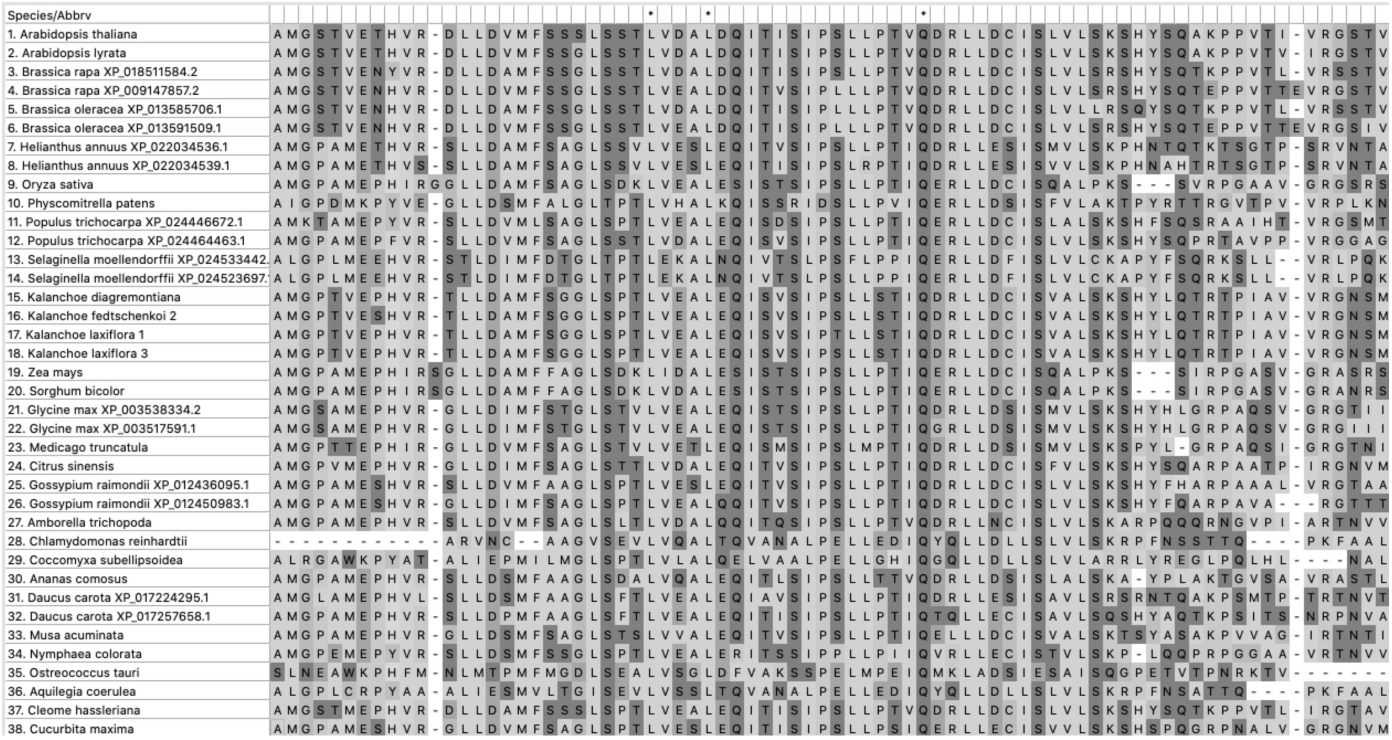

B

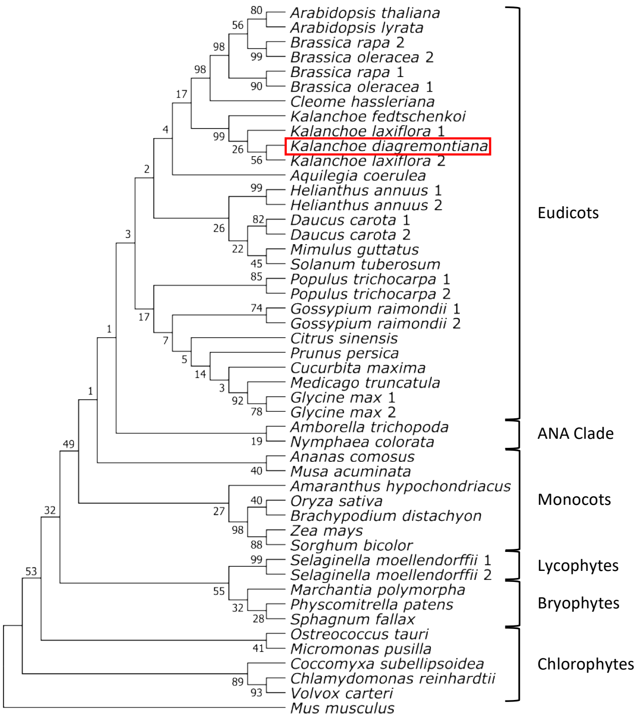

Supplemental Figure S2. Alignment and phylogeny of KdTOR with divergent plant species. A, Amino acid alignment of the 276 bp sequenced fragment of *K. daigremontiana* with divergent eukaryotic species. Positions highlighted with asterisks are conserved. B, Maximum likelihood tree with a Jones-Taylor-Thornton (JTT) model and Gamma (G) substitution rate constructed from an alignment of 92 amino acid sequences from 39 eukaryotic species, including *KdTOR* (red box). Bootstrap values out of 500 replicates are shown as a percentage at each node.

Supplemental Table S1. Sampling strategy for TOR phylogenetic tree construction. TOR sequences from across the land plant phylogeny were selected. The table includes the number of genes in each species and their accessions, the databases used and the dates they were accessed.

Species	Common Name	Source	Access Date	#TOR	Accession	
					TOR1	TOR2
Amaranthus hypochondriacus	prince's feather	Phytozome - Genome	22/05/2020	1	AHYPO_005215-RA	x
Amborella trichopoda	early flowering plant	NCBI Ref Seq RNA	21/05/2020	1	XP_020531683.1	x
Ananas comosus	pineapple	NCBI Ref Seq RNA	21/05/2020	1	XP_020086675.1	x
Aquilegea coerulea	colorado blue columbine	Phytozome - Genome	22/05/2020	1	Aqcoe6G161300.1	x
Arabidopsis lyrata	thale cress	NCBI Ref Seq RNA	21/05/2020	1	XP_020866690.1	x
Arabidopsis thaliana	thale cress	NCBI Ref Seq RNA	21/05/2020	1	NP_175425.2	x
Brachypodium distachyon	false brome grass	NCBI Ref Seq RNA	21/05/2020	1	XP_003568625.2	x
Brassica oleracea	broccillii, cauliflower, cabbage etc	NCBI Ref Seq RNA	21/05/2020	2	XP_013585706.1	XP_013591509.1
Brassica rapa	turnips, chinese cabbage etc	NCBI Ref Seq RNA	21/05/2020	2	XP_018511584.2	XP_009147857.2
Chlamydomonas reinhardtii	chlorophyte algae	NCBI Ref Seq RNA	21/05/2020	1	XP_001697578.1	x
Citrus sinensis	orange	NCBI Ref Seq RNA	21/05/2020	1	XP_006486869.1	x
Cleome hasleriana	spider flower	NCBI Ref Seq RNA	21/05/2020	1	XP_010557537.1	x
Coccomyxa subellipsoidea	chlorophyte algae	NCBI Ref Seq RNA	21/05/2020	1	XP_005644898.1	x
Cucurbita maxima	winter squash	NCBI Ref Seq RNA	21/05/2020	1	XP_022972503.1	x
Daucus carota	wild carrot	NCBI Ref Seq RNA	21/05/2020	2	XP_017224295.1	XP_017257658.1
Glycine max	soybean	NCBI Ref Seq RNA	21/05/2020	2	XP_003538334.2	XP_003517591.1
Gossypium raimondii	cotton	NCBI Ref Seq RNA	21/05/2020	2	XP_012436095.1	XP_012450983.1
Helianthus annuus	sunflower	NCBI Ref Seq RNA	21/05/2020	2	XP_022034536.1	XP_022034539.1
Kalanchoe fedtschenkoi	kalanchoe	Phytozome - Genome	22/05/2020	2	Kaladp0047s0074.1	Kaladp0058s0459.1
Kalanchoe laxiflora	kalanchoe	Phytozome - Genome	22/05/2020	2	Kalax.0075s0076.1	Kalax.0039s0187.1
Marchantia polymorpha	Liverwort	Phytozome - Genome	22/05/2020	1	Mapoly0047s0119.1	x
Medicago trunculata	legume	NCBI Ref Seq RNA	21/05/2020	1	XP_003610644.2	x
Micromonas pusilla	green algae	NCBI Ref Seq RNA	21/05/2020	1	XP_003063087.1	x
Mimulus guttatus	monkeyflower	NCBI Ref Seq RNA	21/05/2020	1	XP_012836833.1	x
Musa acuminata	banana	NCBI Ref Seq RNA	21/05/2020	1	XP_009395017.1	x
Nymphaea colorata	water lily	NCBI Ref Seq RNA	21/05/2020	1	XP_031481229.1	x
Oryza sativa	rice	NCBI Ref Seq RNA	21/05/2020	1	XP_015639567.1	x
Ostreococcus tauri	chlorophyte algae	NCBI Ref Seq RNA	21/05/2020	1	XP_022838330.1	x
Physomitrella patens	moss	NCBI Ref Seq RNA	21/05/2020	1	XP_024379368.1	x
Populus trichocarpa	california poplar	NCBI Ref Seq RNA	21/05/2020	2	XP_024446672.1	XP_024464464.1
Prunus persica	peach	NCBI Ref Seq RNA	21/05/2020	1	XP_020425391.1	x
Selaginella moellendorffii	spikemoss	NCBI Ref Seq RNA	21/05/2020	2	XP_024533442.1	XP_024523697.1
Solanum tuberosum	potato	NCBI Ref Seq RNA	21/05/2020	1	XP_006346275.1	x
Sorghum bicolor	sorghum	NCBI Ref Seq RNA	21/05/2020	1	XP_021303451.1	x
Sphagnum fallax	bog moss	Phytozome - Genome	22/05/2020	1	Sphfalx0024s0121.1	x
Volvox carteri	multicellular chlorophyte algae	NCBI Ref Seq RNA	21/05/2020	1	XP_002954630.1	x
Zea mays	maize	NCBI Ref Seq RNA	21/05/2020	1	NP_001105293.2	x

Supplemental Table S2: List of primers used for gene cloning

	Module	Insert		Primer	Size (bP)
Promoter	*p35SKd*	*35S CaMV* promoter	Forw	gtggtctca **GGAG** GCTAGAGCAGCTTGCCAAC	833
			Rev	gtggtctca **CACC** GGTCGATCGACAGATCTGCG	
	pKdTOR	~1.5 kb upstream of *K. daigremontiana TOR*	Forw	gtggtctca **GGAG** AGGTGAATTTCAAAGCAATCATGC	1467
			Rev	gtggtctca **CATT** CCGATAAACCCTAACTCGATTCC	
Coding Seq	*GUS*	*ß-Glucuronidase* (*GUS*) Reporter	Forw	gtggtctca **AATG** TTACGTCCTGTAGAAACCCCAA	1810
			Rev	gtggtctca **AAGC** TCATTGTTTGCCTCCCTGCT	
	KdTORa	*K. daigremontiana TOR* exon 8 (antisense)	Forw	gtggtctct **AAGC** GGCAATGGGACCTACGGTAG	360
			Rev	gtggtctct **GGTG** GTTGTATGAGGGCAGGACCAC	
Terminator	*Nos Term*	*Nopaline Synthase* Terminator	Forw	gtggtctct **GCTT** GATGATCCCCGATCGTTCAAAC	279
			Rev	gtggtctct **AGCG** GACAGGAGGCCCCGATCTAG	
	35S Term	*35S CaMV* Terminator	Forw	gtggtctct **GCTT** GGGACTCTGGGGTTCGGATC	237
			Rev	gtggtctct **AGCG** GGTGATCTGGATTTTAGTACTGG	

Supplemental Table S3: List of primers used for genotyping and qRT-PCR

**Target**	**Primer Name**	**Sequence 5'→ 3'**
**M13**	M13 Forw	GTTTTCCCAGTCACGAC
	M13 Rev	CAGGAAACAGCTATGAC
NPTII	NPTII Forw	CACAACAGACAATCGGCTGC
	NPTII Rev	GCACGAAGCGGTCAG
***KdTOR* Exons 35-36**	qKdTORForw	CGACGCTGGTGAAACTCTTG
	qKdTORRev	CTTGAGGCAGTCCGTGAAAC
KdGAPDH	qKdGAPDH1	GGAGCAGAGATAACAACCTTC
	qKdGAPDH2	TCCATTCATCAACACAGACTAC
Kd18S	qKd18S Forw	AGAAACGGCTACCACATCCAAG
	qKd18S Rev	GACTCATTGAGCCCGGTATTGT
KdSTM (AT1G62360)	qKdSTM Forw	GGATCAGTTCATGGAGGCTTAC
	qKdSTM Rev	CTTGAACTGGGACTCAATCCTC
KdLEC1 (AT1G21970)	qKdLEC1 Forw	GTCGGAGTATATCGGCTTCATC
	qKdLEC1 Rev	TGTATCGGTGCAGGTACAGAGT
KdS5 (AT3G57490)	qKdS5F1	GACGAGGTGATGAAAATCATGCCG
	qKdS5R1	CGCCACCTCCTTGGAGCAC
KdRAP2 (AT5g13330)	qKdRAP2F1	AGGGGTGAGGCAGAGACCATG
	qKdRAP2R1	CCTTTGAACCTGAGTGCAGCTTCATC
KdPCNA (AF083220)	qKdPCNA1F1	CGAGATGAATGAGCCTGTGTCGC
	qKdPCNA1R1	CTCCGCAATCTTGTACTCCACGAC
KdEBP (AT3g51800)	qKdEBPF1	TTCCCTACATGCATCTCGGTGAAC
	qKdEBPR1	GAGTATGTGCAACCACAGCGATG
KdJAG (AY465924.1)	qKdJAGF1	CCGAACGGAGGGTTTCACCAATC
	qKdJAGR1	GATGGATACAAGTAGGGCTGATGTGGG
KdCUC2 (AT5g53950)	qKdCUC2F2	CAACCGACACCATCAGGAAGTAAGC
	qKdCUC2R2	GGGAAGTCGGAGGATATGTAACTGGC

GenBank accession number of Arabidopsis ortholog in parenthesis.
